# Intraplaque Stretch in Carotid Atherosclerotic Plaque – an Effective Biomechanical Predictor for Subsequent Cerebrovascular Ischemic Events

**DOI:** 10.1371/journal.pone.0061522

**Published:** 2013-04-23

**Authors:** Zhongzhao Teng, Umar Sadat, Wenkai Wang, Nasim S. Bahaei, Shengyong Chen, Victoria E. Young, Martin J. Graves, Jonathan H. Gillard

**Affiliations:** 1 University Department of Radiology, University of Cambridge, Cambridge, United Kingdom; 2 Department of Engineering, University of Cambridge, Cambridge, United Kingdom; 3 Department of Surgery, Cambridge University Hospitals NHS Foundation Trust, Cambridge, United Kingdom; 4 College of Computer Science and Technology, Zhejiang University of Technology, Hangzhou, China; University of Cambridge, United Kingdom

## Abstract

**Background:**

Stretch is a mechanical parameter, which has been proposed previously to affect the biological activities in different tissues. This study explored its utility in determining plaque vulnerability.

**Methods:**

One hundred and six patients with mild to moderate carotid stenosis were recruited in this study (53 symptomatic and 53 asymptomatic). High resolution, multi-sequence magnetic resonance (MR) imaging was performed to delineate various plaque components. Finite element method was used to predict high stretch concentration within the plaque.

**Results:**

During a two-year follow-up, 11 patients in symptomatic group and 3 in asymptomatic group experienced recurrent cerebrovascular events. Plaque stretch at systole and stretch variation during one cardiac cycle was greater in symptomatic group than those in the asymptomatic. Within the symptomatic group, a similar trend was observed in patients with recurrent events compared to those without.

**Conclusion:**

Plaques with high stretch concentration and large stretch variation are associated with increased risk of future cerebrovascular events.

## Introduction

During the last decade we have witnessed a revolution in the imaging-based assessment of atheromatous plaques. Magnetic resonance (MR) imaging has emerged as a non-invasive, non-ionizing imaging technique, which has potential to identify and differentiate between vulnerable and non-vulnerable plaques [1]. While most studies initially used MR for morphological and functional assessment of plaques, there has been a continued effort towards MR-based biomechanical investigation of the diseased vessel [2] because plaque rupture likely occurs if mechanical loading within the fibrous cap (FC) due to blood pressure and flow exceeds its material strength. The superiority of patient-specific biomechanics originates from its inherent capability to integrate information of plaque architecture and associated mechanical conditions. In the only longitudinal study of its kind, we have previously reported that high structural stresses are associated with subsequent cerebrovascular ischemic events [3]. Higher predictive power of biomechanical modelling, compared to morphology alone, was evident from this study. Quantification of structural stresses, however, requires complex computational modelling. A directly measurable biomechanical predictor would, therefore, be a desirable substitute for daily clinical practice. Plaque stretch has shown promise in preliminary studies [4], [5], [6], [7], [8]. With this perspective, here we explore the relationship of plaque stretch, determined by MR-based patient-specific computational modelling, with subsequent cerebrovascular ischaemic events.

## Materials and Methods

**Figure 1:**
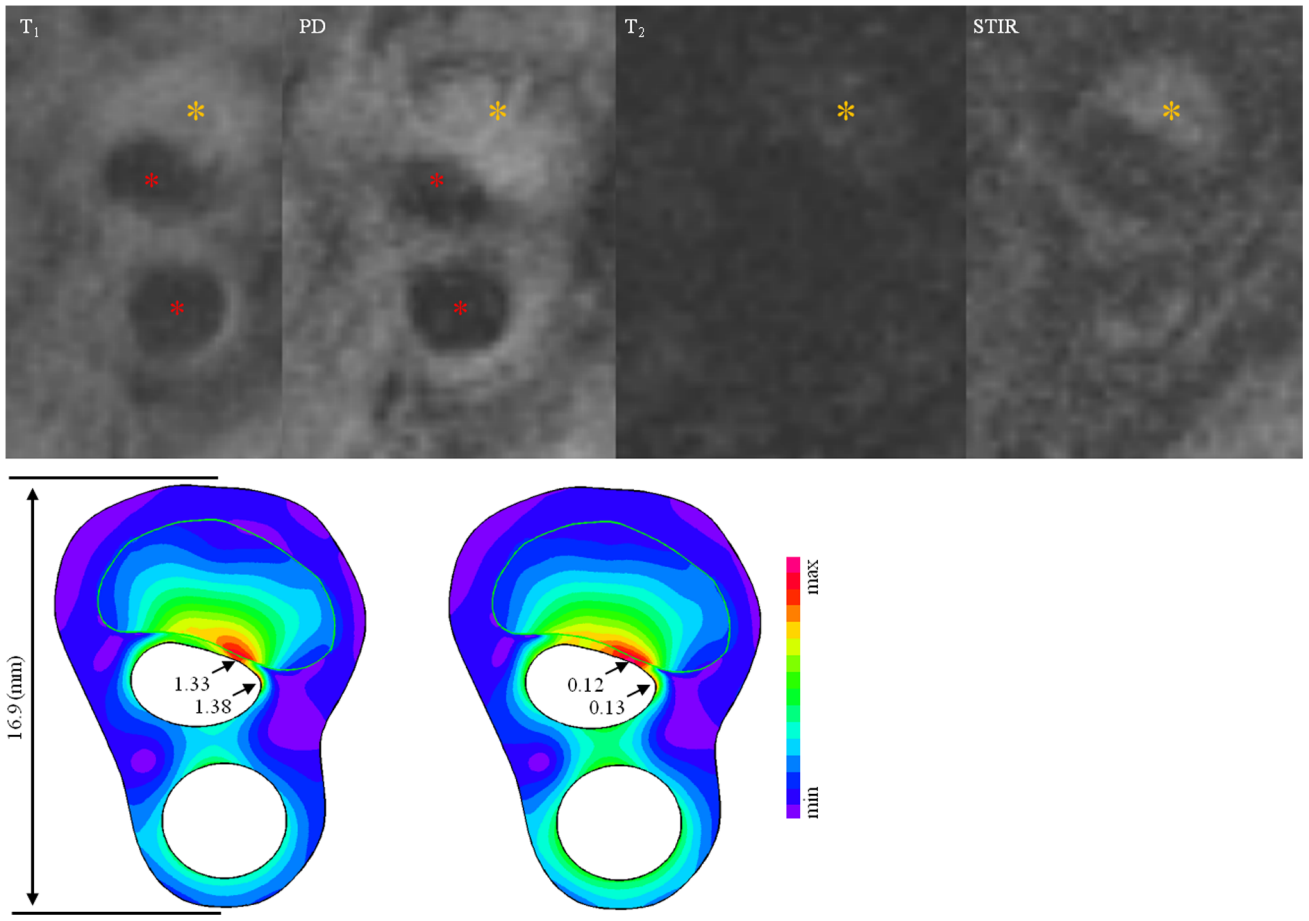
In vivo MR images and band plot of computational results [The 1^st^ row: Signal intensity of a plaque near the carotid bifurcation under T_1_, PD, T_2_ and STIR MR sequences (red asterisks stand for lumen and the brown for fresh intraplaque haemorrhage); the 2^nd^ row, the left image is the band plot of Stretch-P_1_ at systole and the right one is the variation of Stretch-P_1_ during one cardiac cycle].

One hundred and six patients were recruited in this study with fifty-three subjects being acutely symptomatic (i.e. had undergone MR imaging within 72 hours of the acute event) and 53 being asymptomatic (i.e. had never had any events or were asymptomatic over 6 months before undergoing MR imaging). These patients were clinically followed up for 2 years. The protocol was reviewed and approved by the regional research ethics committee (Addenbrooke's Hospital Ethics Committee) and all patients gave written informed consent. The criteria for inclusion were: (1) internal carotid artery (ICA) stenosis of ≥30–69% on duplex imaging during screening assessment; (2) the quality of MR image was rated by using a previously published five-point scale based on noise-to-signal ratio [9], [10] and image quality ≥4 were included for quantitative analysis; and (3) normal heart rhythm, confirmed by 24 hour Holter monitoring and normal transthoracic echocardiography in patients where a cause of stroke other than carotid artery disease was suspected. Exclusion criteria included: (1) cardiac arrhythmias; (2) known coagulation/clotting disorder responsible for patient's symptoms; (3) patients undergoing thrombolysis following the acute cerebrovascular event; and (4) clinical contraindications to MR, e.g. inner ear implants, pacemaker, etc. The clinical end point of the study was a cerebrovascular event including stroke or transient ischaemic attack (TIA) in the region supplied by the index carotid artery, or operation on the index artery. The date of the clinical event was ascertained by review of hospital records and confirmed by patient interviews. Following the event, it was ensured that patients were on the best medical therapy i.e. anti-platelets, cholesterol lowering and antihypertensive medications (if required). The patient demographics are shown in Table 1. Based on previous published protocol [11], multi-contrast weighted images were performed (the 1^st^ row in Fig. 1). Plaque components such as FC, lipid-rich necrotic core (LRNC) and plaque haemorrhage (PH) were manually delineated using CMRTools (London, UK). In total, ten patients in the symptomatic group were excluded for analysis due to various reasons, such as poor image quality and claustrophobia.

**Table 1:** Patient demography.

	Symptomatic (n = 43)	Asymptomatic (n = 53)	p value
Male, n(%)	25 (78.1)	36 (67.9)	0.400*
Age (Mean±SD)	73.7±11.2	69.7±11.1	0.051‡
Diastolic pressure (Mean±SD; mmHg)	79.9±13.1	77.8±14.7	0.349‡
Systolic pressure (Mean±SD; mmHg)	142.3±23.6	138.9±21.1	0.309‡
Heart Rate (Median[IQR])	78 [69, 82]	76 [70, 81]	0.072†
Hypertension, n(%)	33 (76.7)	42 (79.2)	0.808*
Diabetes, n(%)	4 (9.3)	7 (13.2)	0.749*
Atrial fibrillation	7 (16.3)	5 (9.4)	0.363*
Ischaemic heart disease, n(%)	17 (39.5)	19 (35.8)	0.833*
Peripheral vascular disease, n(%)	5 (11.6)	8 (15.1)	0.767
Previous TIA/Stroke, n(%)	10 (23.3)	27 (50.9)	0.007*
Aspirin used before recruitment, n (%)	18 (41.9)	37 (69.8)	0.007*
Stenosis %, (Median[IQR]) §	49 [42, 54]	50 [43, 59]	0.464†
Days of follow-up, (Median[IQR])	428 [94, 689]	659 [385, 830]	0.002†

* Two-sided p value using Fisher's exact test.

‡ Two-tailed p value using Student t test.

† Two-tailed p value using Mann-Whitney test.

§ ECST defined stenosis.

### Finite Element Analysis

The plaque geometry reconstruction was based on the MR segmentation. The rupture FC was recovered using cubic spline function. Under physiological condition, the artery is pressurized, thus circumferential shrinking was applied to generate the start shape for the computational simulation [12]. The plaque components were assumed to be incompressible, piecewise homogeneous, non-linear isotropic and hyper-elastic as described by modified Mooney-Rivlin strain energy density function:
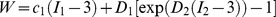
where *I*_1_ is the first stretch invariant and *c*_1_, *D*_1_ and *D*_2_ are material parameters derived from previous experimental work [13], [14], [15], [16]: vessel material, *c*_1_ = 36.8 kPa, *D*_1_ = 14.4 kPa, *D*_2_ = 2; fibrous cap, *c*_1_ = 73.6 kPa, *D*_1_ = 28.8 kPa, *D*_2_ = 2.5; lipid core, *c*_1_ = 2 kPa, *D*_1_ = 2 kPa, *D*_2_ = 1.5; calcification, c_1_ = 368 kPa, *D*_1_ = 144 kPa, *D*_2_ = 2.0. *c*_2_ = 0 for all materials. Rubin et al have performed numerous experiments on determining the relative model-based Young's elastic modulus of thrombi of varying ages using animal models and in humans [15], [17], [18], [19]. Therefore, the material properties of PH were derived from these experiments. For the fresh PH the following parameters were used: *c*_1_ = 1 kPa, *D*_1_ = 1 kPa, *D*_2_ = 0.25. As Rubin et al had categorized thrombi of >1 week age as ‘chronic’, so for MR-identified recent and old PH plaques, the parameters were used: *c*_1_ = 9 kPa, *D*_1_ = 9 kPa, *D*_2_ = 0.25. The stretch of each atherosclerotic component is governed by kinetic equations as:
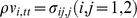
where [*v_i_*] and [*σ_ij_*] are the displacement vector and stress tensor, respectively, *ρ* is the density of each component and *t* stands for time. The pulsatile blood pressure for each patient was measured before MR imaging using tonometry [20], [21] and it was used as the loading condition for the dynamic simulation.

The entire geometric model was meshed using 9-node quadrilaterals and both displacement and strain were assumed to be large. There was no relative movement at the interface of atherosclerotic components. The relative energy tolerance was set to be 0.005. The loading at the outer boundary was set to be zero and two adjacent points were fixed to prevent rigid body displacement. Maximum principle stretch (Stretch-P_1_) was computed using finite element method in ADINA8.6.1 (ADINA R&D, Inc., USA). Stretch-P_1_ can be understood as the ratio of the deformed length and the original. Stretch-P_1_ within the plaque was assessed as well as its variation during one cardiac cycle (the 2^nd^ row in Fig. 1). The stretch variation is defined as,
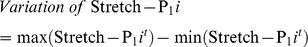
in which the subscript *i* stands for the *i* ^th^ integration node and the superscript *t* stands for time.

Researchers that carried out the mechanical analysis were blinded to the process of image segmentation and the information of patient symptom to avoid subjective bias.

### Statistical Analysis

Normal distribution was tested by Shapiro-Wilk test. For non-normal data, two-tailed Mann-Whitney test and for normal data two-tailed student t test were used. Usually about 8–12 MR slices were taken for each plaque that led to multiple mechanical measurements for a single plaque. The linear mixed effect model was, therefore, used to assess and compare the stretch concentration in different patient groups. This statistical model considered both random effect from slices of an individual plaque and fix effect of different symptom group. Categorical variables were analysed using two-sided Fisher's exact test. Statistical analysis was performed in R 2.10.1 (The R Foundation for Statistical Computing). Statistical threshold was set as p value<0.05.

## Results

In total, 709 slices from 96 patients were analysed and the number of elements and nodes of each slice were 3,029 [2,239, 5,069] and 12,516 [9,354, 20,676] (Median [inter quartile range]), respectively. During the follow-up, 11 (25.6%) patients in the symptomatic group and 3 (5.7%) in the asymptomatic experienced ischaemic cerebrovascular events (p = 0.008). The symptomatic group is further divided into non-recurrent (did not experience ischaemic cerebrovascular events during the follow-up period) and recurrent subgroups (experienced ischaemic cerebrovascular events during the follow-up period). Considering the low incidence in the asymptomatic group, similar analysis is not performed.

### Morphological and Compositional Features

FC rupture and PH had a higher prevalence in symptomatic group than asymptomatic group (FC rupture: 67.4% vs. 22.6%, p<0.001; PH: 62.8% vs. 41.5%, p = 0.043), while there was no significant difference regarding large LRNC (30.2% vs. 50.9%, p = 0.061). Within the symptomatic group, PH was less prevalent in non-recurrent than in recurrent group (53.1% vs. 90.9%, p = 0.033), but FC rupture and large lipid core were both comparable between these two subgroups (FC rupture: 59.4% vs. 90.9%, p = 0.071; large lipid core: 28.1% vs. 36.4%, p = 0.709). These features in detail are listed in Table 2.

**Table 2:** Plaque compositional and morphological features.

	Asymptomatic (n = 53)	No-Recurrent (n = 32)	Recurrent (n = 11)
Fibrous cap rupture, n(%)	12 (22.6)	19 (59.4)	10 (90.9)
Presence of haemorrhage, n(%)	22 (41.5)	17 (53.1)	10 (90.9)
Large lipid core, n(%)*	27 (50.9)	9 (28.1)	4 (36.4)

* Lipid area ≥25% plaque area.

### High Risk Nature of High Stretch Concentration

**Figure 2:**
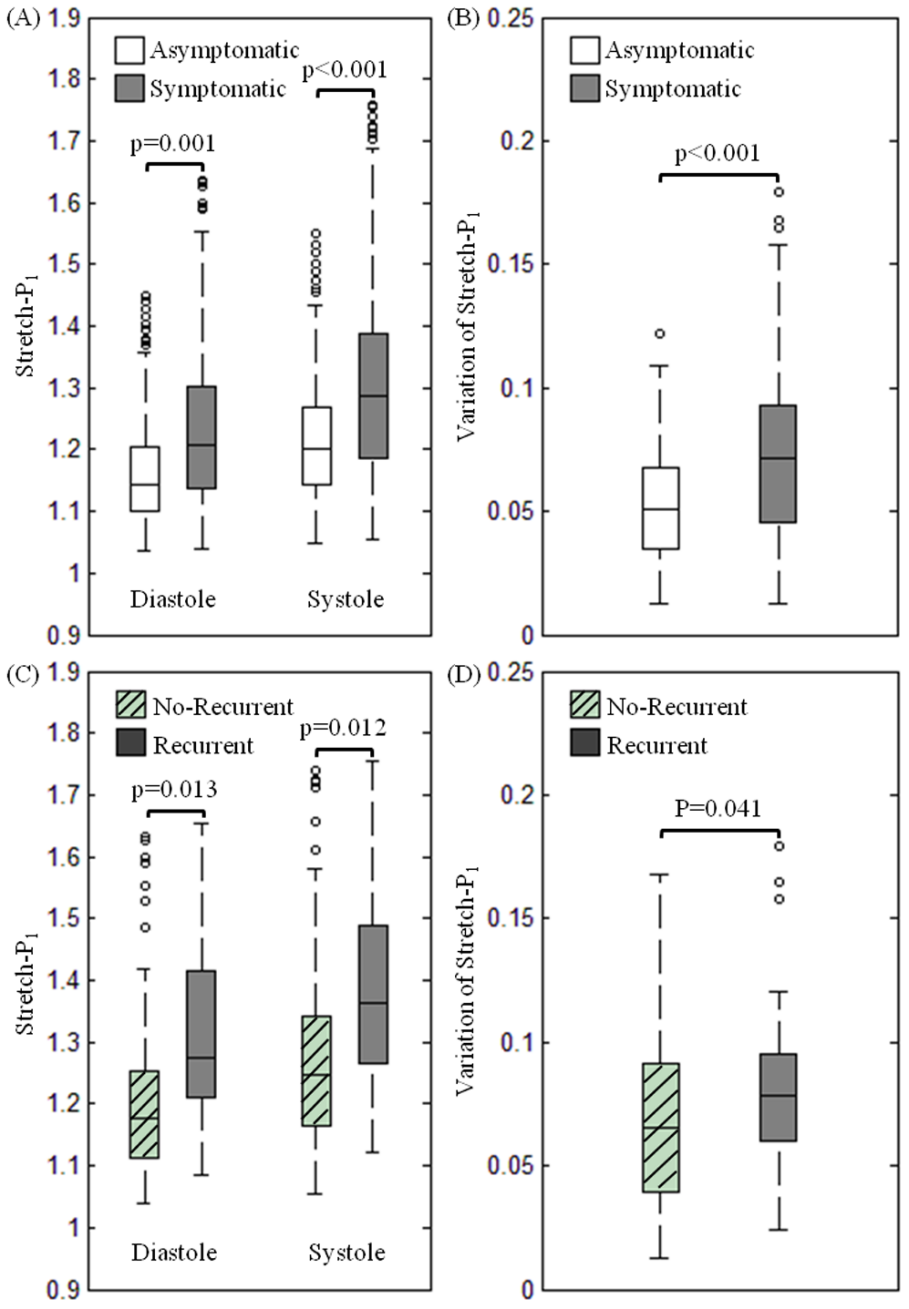
The comparison of plaque stretch (Stretch-P_1_) and its variation in different patient groups (A: Stretch-P_1_ at diastole and systole of asymptomatic and symptomatic patients; B: the variation of Stretch-P_1_ during one cardiac cycle of asymptomatic and symptomatic patients; C: Stretch-P_1_ at diastole and systole of patients in subgroups of with and without experiencing recurrent ischaemic cerebrovascular events; D: the variation of Stretch-P_1_ during one cardiac cycle of patients in these two subgroups).

As shown in Fig. 2A, the stretch levels at both diastole and systole in the asymptomatic group were significantly lower than those in the symptomatic group (median [inter quartile range]; Diastole: 1.145 [1.101, 1.204] vs. 1.209 [1.137, 1.303], p = 0.001; Systole: 1.200 [1.144, 1.267] vs. 1.286 [1.186, 1.386], p<0.001). Variation of blood pressure during each cardiac cycle leads to significant changes in the stretch within the carotid atherosclerotic plaque. Fig. 2B demonstrates the difference of stretch variation during one cardiac cycle between asymptomatic and symptomatic patients (0.071 [0.046, 0.093] vs. 0.051 [0.034, 0.068], p<0.001).

More interestingly, as shown in Fig. 2C, within symptomatic patient group, plaques responsible for recurrent events underwent a much bigger stretch at both diastole and systole compared with those remained stable during the follow-up period (Diastole: 1.275 [1.211, 1.400] vs. 1.177 [1.114, 1.253], p = 0.013; Systole: 1.363 [1.269, 1.475] vs. 1.246 [1.164, 1.342], p = 0.012). Similarly, as shown in Fig. 2D, during one cardiac cycle, the variation of Stretch-P_1_ of unstable lesions was much bigger than the stable ones (0.078 [0.061, 0.095] vs. 0.066 [0.040, 0.091], p = 0.041).

### Proportional Hazard Ratio Analysis of Symptomatic Patients

The hazard ratios (HR) of patient demographics, co-morbidities, plaque disease characteristics and critical stretch conditions in the symptomatic group were listed in Table 3. Both FC rupture (HR = 6.02, p = 0.031) and presence of haemorrhage (HR = 8.00, p = 0.010) were confirmed to be high-risk features associated with recurrent ischaemic cerebrovascular events. The absolute values of Stretch-P_1_ at both diastolic (HR = 9.08, p = 0.025) and systolic (HR = 7.56, p = 0.021) appeared to be risk factors for the recurrent events, as is the variation of Stretch-P_1_ (HR = 31.08, p = 0.045) during a single heartbeat.

**Table 3:** Univariate Cox regression analysis of patient demographics, co-morbidities, carotid artery disease characteristics and critical mechanical conditions in the symptomatic group.

	Hazard ratio	95% Confident interval	p value
Age	0.98	[0.94, 1.01]	0.296
Hypertension	1.76	[0.37, 8.35]	0.474
Diabetes mellitus	0.60	[0.13, 2.60]	0.500
Peripheral vascular disease	0.66	[0.08, 5.24]	0.696
Coronary artery disease	1.42	[0.18, 11.41]	0.739
ECST defined luminal stenosis	1.00	[0.93, 1.06]	0.886
Large lipid core	1.50	[0.44, 5.17]	0.529
Fibrous cap rupture	6.02	[0.77, 47.31]	0.031*
Haemorrhage	8.00	[1.02, 62.86]	0.010*
Stretch-P_1_ at diastole	9.08	[1.32, 62.31]	0.025*
Stretch-P_1_ at systole	7.65	[1.35, 43.32]	0.021*
Variation of Stretch-P_1_	31.08	[4.01, 965.11]	0.045*

## Discussion

To authors' best knowledge, this is the first study assessing the clinical significance of plaque stretch by tracing patient symptoms. It showed that carotid atherosclerotic plaques in symptomatic patients undergo more profound plaque stretch and stretch variation compared to those in asymptomatic patients. It also showed that this observation was more prevalent in plaques responsible for recurrent ischaemic cerebrovascular events than stable lesions. The median value (0.078) of Stretch-P_1_ variation in recurrent group implied that the change of stretch ratio could be up to 8% in some areas in the plaque during one heartbeat. Further analysis indicated that the area with stretch variation above this level could be as big as 4.20±3.98 mm^2^ in the recurrent subgroup.

In this study, most (87.5%) of peak stretch were located within LRNC and PH and only 12.5% of peak stretch were located in FC along lumen region. Understanding the pathological impact of big stretch within the plaque structure would provide support for its applications. Many studies have shown pathological responses on cellular and genetic levels to local stretch within plaques. Pathological stretch can dysregulate cytoskeletal gene expression [22], affecting cell attachment and encouraging programmed cell death [23] and therefore preventing healing in the carotid plaque following acute events [24]. Elevated intraplaque stretch level could lead to smooth muscle cell hypertrophy/hyperplasia by increasing inositol phosphate metabolism and proto-oncogenes expression [25], [26] and possibly promote plaque progression. Moreover, big intraplaque stretch might lead to the rupture of neovessels resulting in the formation and expansion of intraplaque hemorrhage, thereby increasing plaque vulnerability [27].

With this relevance, increasing attentions have been attracted by plaque stretch to assess its clinical significance. It has been shown that the maximum discrepant surface velocity, defined as the maximum of differences between maximum and minimum surface velocities, in symptomatic plaque was significantly higher than that of asymptomatic plaque [28], and mobile plaque (bigger movement during a cardiac cycle) is associated with increased risk of ischaemic stroke [29]. It was found that ‘complex’ plaques follows a specific pattern of reduced radial strain along the longitudinal direction that associated with an outer remodeling, which might be a feature of high-risk plaques [30]. Symptomatic plaques presented both FC defect (erosion, rupture and ulceration) and haemorrhage could be in an extremely high risk due to big stretch concentrations around the ruptured region [24]. It is worth to point out that the ‘stretch’ mentioned in different studies may mean different mechanical determinant, including rigid body displacement, radial strain, maximum principal stretch, etc.

As been reported, stress is an important mechanical parameter, which can be used to assess plaque vulnerability [3], [31], [32], [33], [34], [35], [36], [37]. However, obtained results in this study suggest that stress and stretch might need to be both considered since under most of situation they are located at different locations and they may have different pathological impact. It needs to be pointed out that to obtain the stress distribution within the plaque, apart from the anatomical information, the material properties of each plaque component are required. These material properties however, are not measurable using the current non-invasive techniques. Moreover, it also requires specialised finite element analysis to achieve the distribution of stress. Comparatively, stretch can be potentially measured by image-based ultrasound elastography [4], [5], [6], [7], [8] with more convenience in clinical practice. However, the radio frequency data can only reflect the displacement along the ultrasound beam. Therefore, the strain image produced by current ultrasound elastography is a radial strain map limited the direction of ultrasound beam. Further studies are needed to explore the possibility of measuring the maximum principal stretch using ultrasound radio frequency.

**Figure 3:**
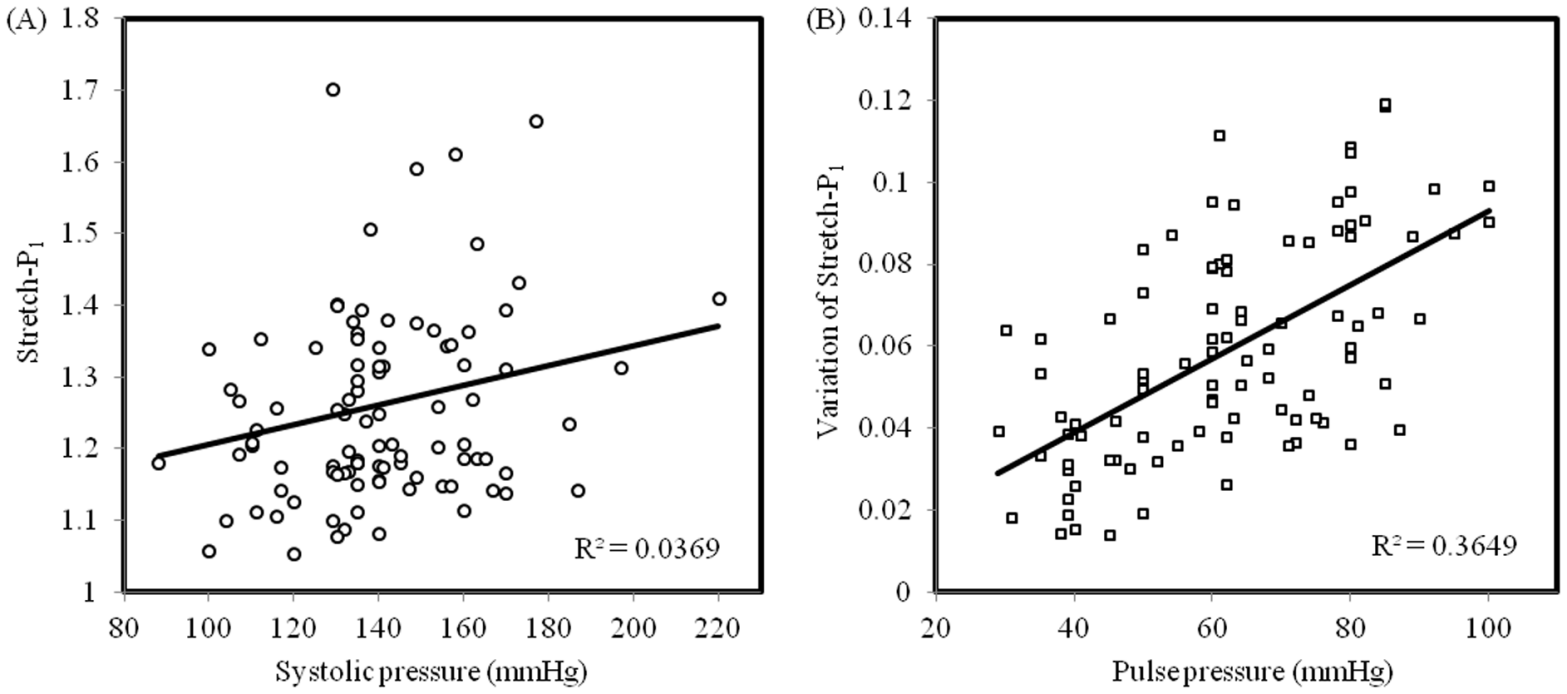
The correlation between Stretch-P_1_ and its variation during one cardiac cycle with systolic blood pressure (A) and pulse pressure (B), respectively.

This study also provides an insight into underlying mechanism of current medical therapies, such as anti-hypertension. Reduction in systolic blood pressure will decrease the stretch level at systole and its variation by narrowing the pulse pressure. The correlation between the Stretch-P_1_ at systole and the systolic blood pressure, however was very weak (correlation coefficient, R^2^ = 0.03), as shown in Fig. 3A. Ten percent reduction in systolic blood pressure only led to 1.97±0.81% decrease in the Stretch-P_1_ at systole, but it would reduce the stretch variation by 24.57±5.90%. A strong correlation between the stretch variation and the pulse pressure was observed (Fig. 3B; R^2^ = 0.365).

Despite the interesting findings reported in this study, several limitations exist: (1) Limited by the MR resolution (in-plane resolution: 0.39×0.39 mm^2^), the FC thickness and size of atherosclerotic component might be over-/under-estimated. This issue had been aware by some of authors involved in this study [38], [39]. Inter-observers studies were performed and the uncertainties among inter observers are around one or the subpixel level [38]; (2) Most biological soft tissues, including healthy arterial wall and fibrous cap, are fibre oriented. The pattern of fibre orientation in healthy arterial wall is clear [40]. Although the material properties of atherosclerotic tissues are layer- and direction-dependent [41], [42], however, the distribution and orientation of collagen and elastin in atherosclerotic tissues are less clear and have been least investigated. Moreover, this information cannot be quantified by current MR technology. The anisotropy, therefore, was not considered in this study; (3) Patient-specific material constants of each atherosclerotic component were not used in this study as they were not measurable using current non-invasive approaches. However, stresses and strains within the arterial wall, fibrous plaque, calcified plaque and lipid have low sensitivities for variation in the elastic modulus [43]. Even a ±50% variation in elastic modulus leads to less than a 10% change in stress at the site of rupture; and (4) Due to the short T_2_, calcium is hard to be detected by traditional 2D Turbo MR sequences [44], [45]. Therefore, in this study, the calcium content could be underestimated.

## Conclusion

In this study we have quantified the plaque stretch and highlighted the association between the degree of stretch and its variation during one cardiac cycle with subsequent ischaemic cerebrovascular event in symptomatic patients.
